# A Filtering Method for Identification of Significant Target mRNAs of Coexpressed and Differentially Expressed MicroRNA Clusters

**DOI:** 10.1155/2018/4932904

**Published:** 2018-09-12

**Authors:** Su Yeon Lee, Soo-Yong Shin, Young Jo Yoon, Yu Rang Park

**Affiliations:** ^1^Bioinformatics Team, Samsung SDS, Seoul, Republic of Korea; ^2^Department of Digital Health, SAIHST, Sungkyunkwan University, Seoul, Republic of Korea; ^3^Office of Clinical Research Information, Asan Medical Center, Seoul, Republic of Korea; ^4^Department of Biomedical Systems Informatics, Yonsei University College of Medicine, Seoul, Republic of Korea

## Abstract

MicroRNA (miRNA) binding is primarily based on sequence, but structure-specific binding is also possible. Various prediction algorithms have been developed for predicting miRNA target genes; the results, however, have relatively high levels of false positives, and the degree of overlap between predicted targets from different methods is poor or null. We devised a new method for identifying significant miRNA target genes from an extensive list of predicted miRNA target gene relationships using hypergeometric distributions. We evaluated our method in statistical and semantic aspects using a common miRNA cluster from six solid tumors. Our method provides statistically and semantically significant miRNA target genes. Complementing target prediction algorithms with our proposed method may have a significant synergistic effect in finding and evaluating functional annotation and enrichment analysis for miRNA.

## 1. Introduction

MicroRNAs (miRNAs) are a class of small RNAs that regulate gene expression at the transcript level, protein level, or both [1–4]. miRNAs modulate gene activity and are aberrantly expressed in most types of cancers [5]. Due to their small size and stability, miRNAs can also be measured in biologic fluids such as plasma and serum and can serve as circulating biomarkers [6–9]. In spite of the continuous attempts to identify miRNAs and to elucidate their basic mechanisms of action, little is understood about their biological functions.

Because of the regulatory role of miRNAs and lack of direct functional annotation to miRNAs, functional enrichment methods for miRNAs rely on their target gene's functional annotations [10–12]. For instance, if the target genes of a specific miRNA are significantly enriched with a set of Gene Ontology (GO) terms, it is reasonable to infer that the miRNA is also involved in the same GO annotations. Several studies on miRNAs have used “predicted target-genes' functional annotation-based” miRNA function prediction strategy [13, 14]; these methods, however, are limited in that they do not consider the many-to-many-to-many tripartite network topology among miRNAs, target genes, and GO annotation [15–17]. In our previous work, we proposed three types of measures (miRNA-centric, target gene-centric, and target link-centric) and a novel index for calculating the functional enrichment of miRNA. Among the three measures, the miRNA-centric measurements showed the best performance [18]. We also found that the miRNA's intrinsic properties of multiplicity and cooperability may be correctly modeled by combined hypergeometric distributions.

Most of the miRNA-to-mRNA target links are estimated by prediction algorithms. However, these algorithms generate a relatively high level of false positives [19], and the degree of overlap between predicted targets from different methods is often poor or null [13, 20]. Studies in this field have developed multiple databases with enormous amount of miRNA-to-target mRNA relationships computed using diverse algorithms [21], whereas only a few experimentally validated targets are available [22, 23]. In light of this circumstance, there is an unmet need for a method for identifying a significant miRNA target from a copious amount of predicted miRNA-target mRNA pairs.

According to miRNA characteristics (multiplicity and cooperatively activities), we employed the hypergeometric distribution to identify significant miRNA target genes from the extensive list of miRNA target genes. We also evaluated the performance of our method in two aspects: statistical significance and functional enrichment.

## 2. Methods

### 2.1. Computational Methods

To find significant target mRNAs from the input miRNA cluster, we first searched for target mRNAs from all miRNA members within the input cluster from the miRNA target database. For each targeted mRNAs, we then calculated the numbers of miRNAs that have target relationships (*p*_*i*_) with the mRNA and those that do not (*p*_*j*_) using the two-by-two contingency table. We also calculated the numbers of miRNAs not in the input miRNA cluster by dividing those that have a target relationship (*p*_*k*_) with the mRNA and those that do not (*p*_*l*_), as shown in Table 1. Functional enrichment was tested from this contingency table using a hypergeometric distribution. The hypergeometric distribution applies to sampling without replacement from a finite population whose elements can be classified into two mutually exclusive categories: has/does not have a target relationship.

We then calculated the adjusted *p* values using the Bonferroni correction. Finally, for evaluating our methods, 10,000 simulated mRNA sets of the same size were also randomly sampled from the target mRNAs of the input miRNA cluster.

Using hypergeometric distribution, we assumed that the coordinated function among miRNAs within a cluster is valid when these miRNAs are regulated or annotated by common factors such as same target mRNA, Gene Ontology, or pathway.

### 2.2. Data Set: miRNA Clusters

We obtained an miRNA set created by Volinia et al. [24] that has differentially expressed sets of up- or downregulated miRNAs in six solid tumor samples. Among the miRNA clusters, we selected an miRNA cluster composed of 57 miRNAs by prediction analysis of microarray (PAM) in six solid tumor samples versus normal tissues. The complete list of 57 miRNAs is in Additional File 1.

### 2.3. Creating Variations of the miRNA-mRNA Target Pair

**Figure 1:**
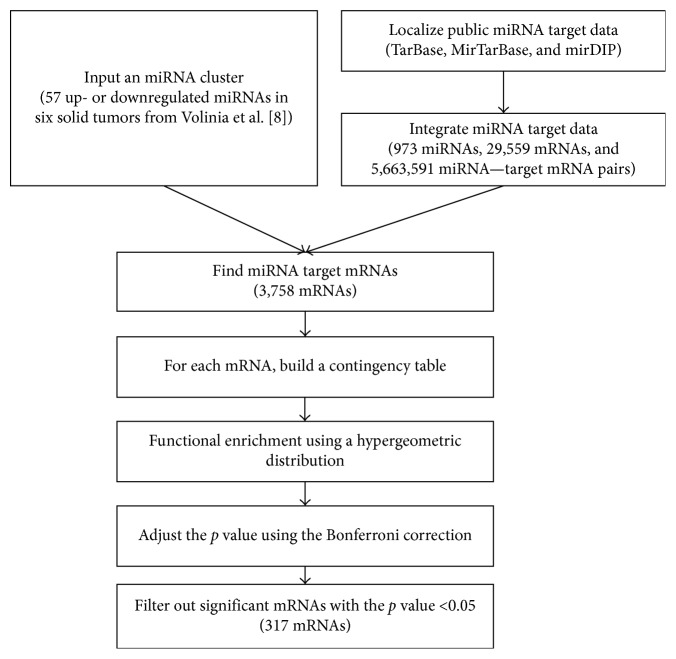
Flowchart of the computational method for identifying significant miRNA target genes.

To build the miRNA-mRNA target pair, we chose three representative miRNA databases: TarBase (Data release 6.0, February 28^th^, 2014) [23], MirTarBase (Data release 4.5, February 28^th^, 2014) [22], and mirDIP (Data release 1.0, February 12^th^, 2014) [14]. The TarBase and MirTarBase databases provide experimentally validated miRNA-target interaction data and evidence level (strong and less strong) of each interaction. The mirDIP database provides *in silico*-predicted miRNA-target interaction data from six established target prediction algorithms and 12 miRNA prediction databases. GO annotation of miRNA-target-mRNA was obtained from the Entrez Gene database. We excluded GO associations with ND (no biological data) and NR (not recorded) evidence code. Detailed processes are provided in Figure 1.

### 2.4. Statistical and Semantic Evaluation Measurement

To evaluate our methods, we compared the performance in terms of statistical significances between a significant 317 mRNA cluster and randomly simulated 10,000 clusters. Each randomly generated cluster had the same size as the significant mRNA set. GO functional enrichment analysis was performed for all mRNA sets using GO annotations retrieved from the NCBI Entrez Gene database. We filtered out 339 GO terms that were greater than 0.05. The resulting lists of 377 GO terms are shown in Additional File 2. To reduce the number of GO terms, enriched GO terms and *p* values were submitted to REduce and Visualize GO (REViGO).

We computed the average log *p* values of ranked GO term sets from functional enrichment analysis of the significant mRNA set and the randomly simulated 10,000 mRNA sets. These average log *p* values were then used for comparing the performance. Functional enrichment was performed using GO annotations of mRNA from NCBI Entrez gene [25]. Average log *p* values of ranked GO terms were based on the general assumption that highly significant GO terms are more desirable because it means the members of the cluster are highly correlated to each other. For semantic evaluation of the significant mRNA set, we used REViGO, which is a web-based system that summarizes a list of GO terms by finding a representative subset of the terms using the semantic similarity-based clustering algorithm [26].

## 3. Results

### 3.1. Statistical Significances

**Figure 2:**
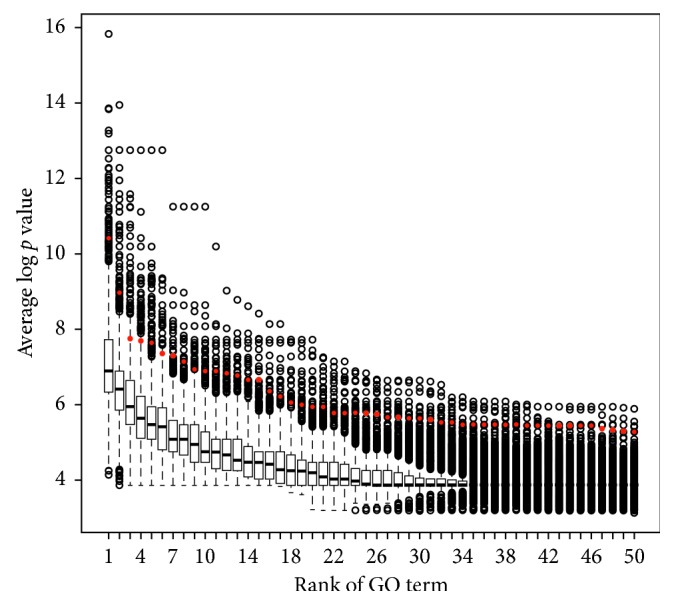
Evaluation of statistical significance across thresholds. The significant mRNA set and randomly simulated 10,000 clusters are shown as red dotted graphs and box plots, respectively.

For evaluating our methods, we compare performance in terms of statistical significances between a significant 317 mRNA cluster and randomly simulated 10,000 clusters. Each randomly generated cluster has the same size with the significant mRNA cluster. All mRNA cluster performed GO functional enrichment analysis using GO annotation from the NCBI Entrez gene.  Figure 2 shows the distributions of average log *p* values for the rank of GO terms which belong to the biological process category. The significant mRNA set is shown as the red dotted graph. Randomly simulated 10,000 clusters are shown as box plots. The significant mRNA set showed a higher average log *p* value than random clusters did, which indicated that the members of the cluster highly correlated and meaningfully composed.

### 3.2. Gene Ontology Analysis of Significant miRNA Target Genes

Using the UniProt database as background and the default semantic measure (SimRel), our analysis clearly showed that biological processes associated with cancer metabolism, regulation of cell death and apoptotic process, and negative regulation of autophagy were significantly overrepresented.

**Figure 3:**
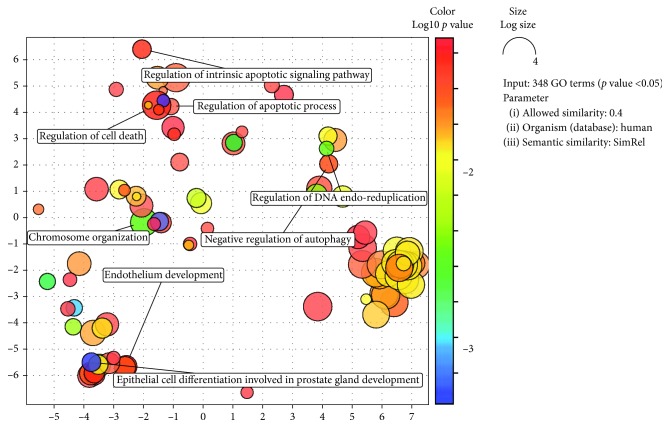
REViGO scatter plot for the significant mRNA set.

Figure 3 shows the REViGO scatter plot represented in a two-dimensional space derived by applying multidimensional scaling to a matrix of GO terms semantic similarities. The resulting lists of 339 GO terms along with their *p* values were further summarized by the REViGO reduction analysis tool that condenses the GO description by removing redundant terms. The remaining terms after the redundancy reduction were plotted in a two-dimensional space. Bubble color indicates the *p* value (legend in the upper right-hand corner): the two ends of the colors are red and blue, which represent lower and higher *p* values, respectively. Size indicates the relative frequency of the GO term in the underlying reference UniProt databases (more general terms are represented by larger size bubbles).

## 4. Discussion

Functional enrichment studies for miRNA expression are performed in three steps: (1) selecting differentially expressed miRNAs, (2) finding their target mRNA, and (3) carrying out analysis of mRNA set overrepresentation [27]. Functional enrichment studies for miRNAs are mostly based on the annotation of target mRNA; however, due to improvements in the miRNA target prediction algorithms, a large number of target mRNAs are predicted. Considering this, filtering out significant mRNAs using a stable statistical method is of great importance. In this study, we proposed a method for identifying the significant miRNA target mRNA from the miRNA cluster. The proposed method was verified by functional enrichment analysis of differentially expressed or coexpressed miRNA clusters.

Inaccurate functional enrichment methods are a hindrance in increasing clinical utility for miRNAs, such as miRNA-based biomarkers or predictors [28, 29]. Several tools have been recently established for direct prediction of miRNA functions [10, 30]; however, these methods do not consider the regulatory or indirect functions of miRNAs, such as regulation or inhibition of target genes [31]. The intrinsic properties of multiplicity and cooperative activities of miRNAs should be considered while annotating the miRNA function. miRGator v3.0 is a tool created considering these characteristics and allows the user to manually select miRNAs and target mRNAs [32]. However, such tools are only useful when the number of miRNA and mRNA pairs is small.

The limitation of the proposed method is that the hypergeometric distribution has a significant effect when members belonging to an miRNA cluster are regulated by common factors such as the target mRNA, GO, and pathway. The proposed method constructs a target mRNA set with statistical significance by receiving miRNA clusters with similar expression characteristics. The assumption of hypergeometric is well suited to this problem because the cluster-received input already has similar characteristics.

The miRNA target prediction algorithms were modified to generate more accurate results based on the expanding understanding of the molecular mechanism of miRNA regulation. Nevertheless, identifying significant target mRNAs from the numerous, uncurated miRNA target links remain as a problem. Our method is based on computationally identifying statistically significant mRNAs using predicted or experimentally validated target relationships. Complementing target prediction algorithms with our proposed method may have significant synergistic effects in finding and evaluating functional annotation and enrichment analysis for miRNA.

## Figures and Tables

**Table 1:** 2 × 2 contingency table of miRNA frequency calculated for each target mRNA.

Target mRNA	Input miRNA cluster	
	In cluster	Not in cluster
Has a target relationship	*p*_*i*_	*p*_*k*_
Does not have a target relationship	*p*_*j*_	*p*_*l*_

## Supplementary Materials

Supplementary 1 Additional File 1: the complete list of 57 miRNAs.

Supplementary 2 Additional File 2: the resulting lists of 377 GO terms.

## References

[1] Bartel D. P. (2009). MicroRNAs: target recognition and regulatory functions. *Cell*.

[2] Nelson P. T., Baldwin D. A., Scearce L. M., Oberholtzer J. C., Tobias J. W., Mourelatos Z. (2004). Microarray-based, high-throughput gene expression profiling of microRNAs. *Nature Methods*.

[3] Lai E. C. (2003). microRNAs: runts of the genome assert themselves. *Current Biology*.

[4] Sun K., Lai E. C. (2013). Adult-specific functions of animal microRNAs. *Nature Reviews Genetics*.

[5] Iorio M. V., Croce C. M. (2017). MicroRNA dysregulation in cancer: diagnostics, monitoring and therapeutics. A comprehensive review. *Embo Molecular Medicine*.

[6] Chen X., Ba Y., Ma L. (2008). Characterization of microRNAs in serum: a novel class of biomarkers for diagnosis of cancer and other diseases. *Cell Research*.

[7] Mitchell P. S., Parkin R. K., Kroh E. M. (2008). Circulating microRNAs as stable blood-based markers for cancer detection. *Proceedings of the National Academy of Sciences of the United States of America*.

[8] Sozzi G., Boeri M., Rossi M. (2014). Clinical utility of a plasma-based miRNA signature classifier within computed tomography lung cancer screening: a correlative MILD trial study. *Journal of Clinical Oncology*.

[9] Belzeaux R., Lin R. X., Turecki G. (2017). Potential use of microRNA for monitoring therapeutic response to antidepressants. *CNS Drugs*.

[10] Ulitsky I., Laurent L. C., Shamir R. (2010). Towards computational prediction of microRNA function and activity. *Nucleic Acids Research*.

[11] Wu Y. Q., Chen D.-J., He H.-B. (2012). Pseudorabies virus infected porcine epithelial cell line generates a diverse set of host microRNAs and a special cluster of viral microRNAs. *PLoS One*.

[12] Xiao Y., Xu C., Guan J. (2012). Discovering dysfunction of multiple microRNAs cooperation in disease by a conserved microRNA co-expression network. *PLoS One*.

[13] Ekimler S., Sahin K. (2014). Computational methods for microRNA target prediction. *Genes*.

[14] Shirdel E. A., Xie W., Mak T. W., Jurisica I. (2011). NAViGaTing the micronome–using multiple microRNA prediction databases to identify signalling pathway-associated microRNAs. *PLoS One*.

[15] Gaidatzis D., van Nimwegen E., Hausser J., Zavolan M. (2007). Inference of miRNA targets using evolutionary conservation and pathway analysis. *BMC Bioinformatics*.

[16] Xu J., Wong C. (2008). A computational screen for mouse signaling pathways targeted by microRNA clusters. *RNA*.

[17] Gusev Y (2008). Computational methods for analysis of cellular functions and pathways collectively targeted by differentially expressed microRNA. *Methods*.

[18] Lee S. Y., Sohn K. A., Kim J. H. (2012). MicroRNA-centric measurement improves functional enrichment analysis of co-expressed and differentially expressed microRNA clusters. *BMC Genomics*.

[19] Hon L. S., Zhang Z. (2007). The roles of binding site arrangement and combinatorial targeting in microRNA repression of gene expression. *Genome Biology*.

[20] Sethupathy P., Megraw M., Hatzigeorgiou A. G. (2006). A guide through present computational approaches for the identification of mammalian microRNA targets. *Nature Methods*.

[21] Ritchie W., Rasko J. E. (2014). Refining microRNA target predictions: sorting the wheat from the chaff. *Biochemical and Biophysical Research Communications*.

[22] Hsu S. D., Lin F.-M., Wu W.-Y. (2011). miRTarBase: a database curates experimentally validated microRNA-target interactions. *Nucleic Acids Research*.

[23] Vergoulis T., Vlachos I. S., Alexiou P. (2012). TarBase 6.0: capturing the exponential growth of miRNA targets with experimental support. *Nucleic Acids Research*.

[24] Volinia S., Calin G. A., Liu C.-G. (2006). A microRNA expression signature of human solid tumors defines cancer gene targets. *Proceedings of the National Academy of Sciences*.

[25] NCBI Resource Coordinators (2014). Database resources of the National Center for Biotechnology Information. *Nucleic Acids Research*.

[26] Supek F., Bošnjak M., Škunca N., Šmuc T. (2011). REVIGO summarizes and visualizes long lists of gene ontology terms. *PLoS One*.

[27] Garcia-Garcia F., Panadero J., Dopazo J., Montaner D. (2016). Integrated gene set analysis for microRNA studies. *Bioinformatics*.

[28] Bleazard T., Lamb J. A., Griffiths-Jones S. (2015). Bias in microRNA functional enrichment analysis. *Bioinformatics*.

[29] Ritchie W., Flamant S., Rasko J. E. (2009). Predicting microRNA targets and functions: traps for the unwary. *Nature Methods*.

[30] Lu M., Shi B., Wang J., Cao Q., Cui Q. (2010). TAM: a method for enrichment and depletion analysis of a microRNA category in a list of microRNAs. *BMC Bioinformatics*.

[31] Lim L. P., Lau N. C., Garrett-Engele P. (2005). Microarray analysis shows that some microRNAs downregulate large numbers of target mRNAs. *Nature*.

[32] Cho S., Jang I., Jun Y. (2013). miRGator v3.0: a microRNA portal for deep sequencing, expression profiling and mRNA targeting. *Nucleic Acids Research*.

